# Letter to the editor on: prophylactic nicotinamide treatment protects from rotenone-induced neurodegeneration by increasing mitochondrial content and volume

**DOI:** 10.1186/s40478-024-01768-1

**Published:** 2024-04-05

**Authors:** Cinzia Bocca, Judith Kouassi-Nzoughet, Juan Manuel Chao de la Barca, Dominique Bonneau, Christophe Verny, Philippe Gohier, Christophe Orssaud, Pascal Reynier

**Affiliations:** 1 Laboratoire de Biochimie et biologie moléculaire, Centre Hospitalier Universitaire (CHU), Angers, France; 2 Unité Mixte de Recherche (UMR) MITOVASC, Institut National de la Santé et de la Recherche Médicale (INSERM U1083), Centre National de la Recherche Scientifique (CNRS 6015), Université d’Angers, Angers, France; 3 Faculté de Pharmacie de Paris, Université Paris Cité, CNRS, CiTCoM, Paris, F-75006 France; 4 Département de Neurologie, Centre Hospitalier Universitaire (CHU), Angers, France; 5 Département d’Ophtalmologie, Centre Hospitalier Universitaire (CHU), Angers, France; 6 Functional Unit of Ophthalmology, Ophtara Rare disease Center, Sensgène Filière, European Hospital Georges Pompidou, ERN EYE, University Hospital Paris Centre, Assistance Publique des Hôpitaux de Paris, Paris, France

**Keywords:** Glaucoma, Leber hereditary optic neuropathy, Mitochondria, Nicotinamide, Optic neuropathy, Retinal ganglion cells, Vitamine B3

Dear editor,

We have read with great interest the article published by Otmani et *al.* in *Acta Neuropathologica Communications* reporting that nicotinamide (vitamin B3) protects retinal ganglion cells against rotenone-induced neurodegeneration [1]. In a previous pre-clinical study, published in 2017 in *Science*, the same team had shown that oral intake of nicotinamide could prevent retinal ganglion cell loss in a murine model of glaucoma [2]. Subsequently, encouraging results of two clinical trials of nicotinamide in individuals affected with glaucoma were reported [3, 4]. In the current study, Otmani et *al.* provide a pre-clinical rationale for the use of nicotinamide in Leber’s Hereditary Optic Neuropathy (LHON), one of the two most common hereditary optic neuropathies.

We would like to provide additional arguments in favor of the use of nicotinamide in optic neuropathies. Using a hypothesis-free metabolomics approach, performed in 2021 on 18 individuals affected with LHON and to 18 healthy controls, we evidenced that individuals with LHON had a nicotinamide deficiency in blood compared to controls [5]. The metabolomic signature in plasma of individuals with LHON comprised 13 discriminating metabolites including dietary metabolites (nicotinamide, taurine, choline, 1-methylhistidine and hippurate), mitochondrial energetic substrates (acetoacetate, glutamate and fumarate) as well as inosine, an essential metabolite for purine biosynthesis.

In addition, we carried out a similar metabolomics investigation in individuals affected with Dominant Optic Atrophy (DOA) caused by *OPA1* pathogenic variants, the other most common form of hereditary optic neuropathy. In this study, we compared the plasma of 25 affected individuals with that of 20 healthy controls [6]. Within the metabolomic signature of DOA, we found, a C_6_H_6_N_2_O chemical compound, not identified at the time of the publication, whose concentration was significantly lowered. Subsequently, this compound was identified as nicotinamide. A deeper analysis taking into account the severity of the DOA phenotype showed that, compared with healthy controls, nicotinamide was lowered by 37% in asymptomatic carriers (*p* < 0.05), by 35% in DOA patients (*p* < 0.001) and by 64% in patients with most severe syndromic form of the disease referred to as DOA+ (*p* < 0.001) (Fig. 1, unpublished data). Altogether, these results give arguments in favor of the therapeutic interest of nicotinamide in both isolated and syndromic forms of DOA.

We therefore have launched a clinical trial (https://classic.clinicaltrials.gov/ct2/show/NCT06007391) to test the safety and efficacy of three grams per day of oral nicotinamide for six months in 25 individuals with DOA who will have ophthalmic, neurological and biological (blood nicotinamide concentration) monitoring.

Finally, primary open-angle glaucoma, was the third disease for which our hypothesis-free metabolomics approach revealed a nicotinamide deficiency in affected individuals compared to healthy controls. This deficiency was confirmed by an independent targeted mass spectrometry measurement on two cohorts of individuals (34 and 30 patients compared to 20 and 15 healthy controls respectively) [7].

There is therefore a deregulation of nicotinamide metabolism in glaucoma, the most common form of optic neuropathy worldwide, as well in LHON and DOA, the two most common hereditary optic neuropathies.

We can wonder whether such deficiency in nicotinamide could be present in other rare and common forms of optic neuropathies, and whether nicotinamide might not universally protect retinal ganglion cells. The origin of this relative deficiency in nicotinamide is undoubtedly not of dietary origin but probably rather the result of a dysfunction of mitochondrial energy metabolism leading to an excessive consumption of nicotinamide used by cells for the endogenous synthesis of Nicotinamide Adenine Dinucleotide (NAD) which plays a major role in this energetic metabolism. Future clinical trials are needed to further characterize the potential of blood nicotinamide concentration as a biomarker for optic neuropathies or, more generally, for pathologies involving mitochondrial dysfunction. Indeed, plasma nicotinamide assay is rarely used in clinical practice, and the most widespread assay using High Performance Liquid Chromatography coupled to ultraviolet detection (HPLC-UV) performed on whole blood, is not very sensitive. The recent use of mass spectrometry in plasma samples should make it possible to detect more subtle variations in nicotinamide concentration that may be of clinical interest. It will also be of great interest to know the results of future clinical trials using nicotinamide in these different forms of optic neuropathies.

**Fig. 1:**
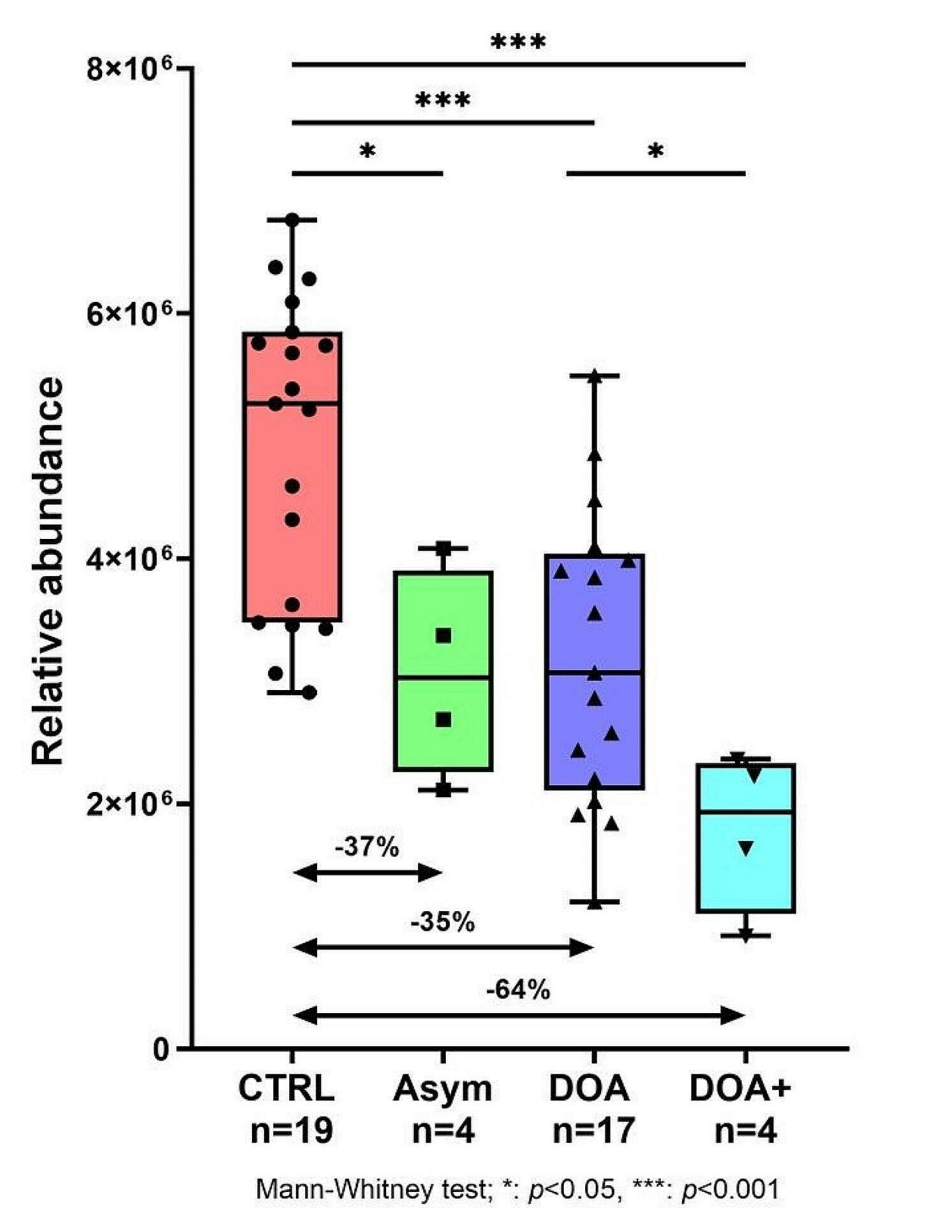
Comparison of nicotinamide level detected after a metabolomic approach [6] in the plasma of healthy controls (CTRL), asymptomatic *OPA1* pathogenic variants carriers (Asym), and patient with isolated (DOA) or syndromic (DOA+) forms. * *p* < 0.05; *** *p* < 0.001

## Data Availability

The datasets discussed in this letter have been published in references 5–8.

## Abbreviations

DOA: Dominant Optic Atrophy
LHON: Leber Hereditary Optic Neuropathy
NAD: Nicotinamide Adenine Dinucleotide
HPLC: High Performance Liquid Chromatography
